# Catastrophic Forgetting in Deep Graph Networks: A Graph Classification Benchmark

**DOI:** 10.3389/frai.2022.824655

**Published:** 2022-02-04

**Authors:** Antonio Carta, Andrea Cossu, Federico Errica, Davide Bacciu

**Affiliations:** ^1^Computer Science Department, University of Pisa, Pisa, Italy; ^2^Scuola Normale Superiore, Pisa, Italy

**Keywords:** continual-learning, lifelong-learning, catastrophic-forgetting, deep-graph-networks, benchmarks

## Abstract

In this work, we study the phenomenon of catastrophic forgetting in the graph representation learning scenario. The primary objective of the analysis is to understand whether classical continual learning techniques for flat and sequential data have a tangible impact on performances when applied to graph data. To do so, we experiment with a structure-agnostic model and a deep graph network in a robust and controlled environment on three different datasets. The benchmark is complemented by an investigation on the effect of structure-preserving regularization techniques on catastrophic forgetting. We find that replay is the most effective strategy in so far, which also benefits the most from the use of regularization. Our findings suggest interesting future research at the intersection of the continual and graph representation learning fields. Finally, we provide researchers with a flexible software framework to reproduce our results and carry out further experiments.

## 1. Introduction

Building a robust machine learning model that incrementally learns from different tasks without forgetting requires methodologies that account for drifts in the input distribution. The Continual Learning (CL) research field addresses the catastrophic forgetting problem (Grossberg, 1980; French, 1999) by devising learning algorithms that improve a model's ability to retain previously gathered information while learning across multiple steps. Each step in a CL scenario constitutes a new learning experience providing new data to the model, whose distribution may be different with respect to the previously encountered ones. As of today, CL methods have been studied from the perspective of flat data (data without a strong temporal or geometrical structure) (Kirkpatrick et al., 2017; Shin et al., 2017; Maltoni and Lomonaco, 2018) and, to a lesser extent, sequential data (Ehret et al., 2020; Sodhani et al., 2020; Cossu et al., 2021). In particular, the literature on CL revolves around three main families of strategies aimed at tackling catastrophic forgetting (Parisi et al., 2019): regularization strategies, architectural strategies and replay strategies. Though not entirely comprehensive, this taxonomy includes most of the currently used CL strategies.

**Regularization strategies** add a penalization to the standard loss function to enforce the stability of existing parameters. For example, the penalization may force parameters deemed important for a specific task not to change much during training (Kirkpatrick et al., 2017), or it may impose stability of the output activations during different tasks through distillation (Li and Hoiem, 2016). **Architectural strategies** try to mitigate forgetting by enhancing the model's plasticity. Typically, they expand the network by adding more units (Marsland et al., 2002; Draelos et al., 2017), an entirely new module (Rusu et al., 2016; Cossu et al., 2020), or by expanding and then compressing the resulting architecture (Hung et al., 2019; Srivastava et al., 2019). Finally, **replay strategies** mix input patterns from the current step with patterns from previously encountered steps (Isele and Cosgun, 2018; Rolnick et al., 2019). Replay memory management is crucial because it is not feasible to store all the patterns from previous steps. Generative replay, instead, overcomes this problem by training a generative model (with fixed space occupancy) that provides on-demand previous patterns (Shin et al., 2017; Wang et al., 2019; van de Ven et al., 2020).

Graph Representation Learning (GRL) is the study of machine learning models that can make predictions about input data represented as a graph. GRL methods naturally find application in social sciences (Nechaev et al., 2018), recommender systems (Bobadilla et al., 2013), cheminformatics (Micheli et al., 2007), security (Iadarola, 2018), and natural language processing (Marcheggiani et al., 2018), where each graph has a potentially different topology (Micheli et al., 2007).

There is a long and consolidated history of works that discuss these problems in static scenarios, where data is completely available from the beginning (Sperduti and Starita, 1997; Frasconi et al., 1998; Micheli, 2009; Scarselli et al., 2009). Nowadays, the models that can process a broad spectrum of graphs by means of local and iterative processing of information are called Deep Graph Networks^1^ (DGNs) (Bacciu et al., 2020). Generally speaking, DGNs propagate nodes' information across the graph by stacking several graph convolutional layers on top of each other. Each layer works by aggregating each node's neighboring information, and it ultimately produces node representations that can be used to make predictions about nodes, links, or entire graphs. For the sake of brevity, we refer the reader to recent works that summarize the state of the art (Bronstein et al., 2017; Battaglia et al., 2018; Bacciu et al., 2020; Wu et al., 2020).

At present, the literature lacks an analysis of catastrophic forgetting in models that deal with graphs. The few existing works focus on new approaches which are not compared to existing CL strategies on challenging benchmarks (Wang et al., 2020; Zhou and Cao, 2021). This work makes the first step in this direction by carrying out continual learning experiments on graph classification benchmarks in a robust and controlled framework. In this context, we investigate whether specific GRL regularization strategies can mitigate catastrophic forgetting by enforcing structural information preservation.

Our contribution is two-fold. First of all, we study whether CL techniques for flat data still work on the graph domain. If that is not the case, the results will call for different and novel approaches to be developed. Secondly, we provide a robust and reproducible framework to carry out Continual Learning experiments on graph-structured data. Indeed the GRL field has suffered serious reproducibility issues that impacted chemical and social benchmarks (Shchur et al., 2018; Errica et al., 2020). By publicly releasing our code and adopting a clear experimental evaluation, we prevent common malpractices such as the usage of custom data splits for model selection and model assessment, the absence of a model selection, and incorrect evaluations of the estimated risk on the validation (rather than test) set.

## 2. Method

We now detail the CL strategies and deep graph networks used to evaluate catastrophic forgetting in the domain of graph-structured data. To the best of our knowledge, this is one of the first studies to investigate this particular aspect. To keep the discussion clear, we will focus on regularization and replay strategies applied to simple architectures for graphs, deferring more complex techniques to future studies.

### 2.1. Continual Learning Strategies

#### 2.1.1. Elastic Weight Consolidation

Elastic Weight Consolidation (Kirkpatrick et al., 2017) is a regularization technique which prevents changes in parameters that are important for previous steps. Formally, EWC adds a squared penalty term R to the classification loss at training time:


(1) $$R(\Theta ,\Omega )=\lambda \sum_{i=1}^{n-1}\Omega_{i}\|\Theta_{i}-\Theta_{n}{\|}_{2}^{2},$$

where Θ_**n**_ is the vector of parameters of current step *n*, Θ_i_ is the vector of parameters from previous step *i* and Ω_**i**_ is the vector of parameter importances for step *i*. The hyperparameter λ controls the trade-off between classification accuracy on current step and stability of parameters. The importance for step *n* is computed at the end of training on step *n*, through a diagonal approximation of the Fisher Information Matrix:


(2) $$\Omega_{n}=E_{(x,y)\in D}\left[\left(\nabla_{\Theta_{n}}\log p_{\Theta_{n}}{\left(y|x\right)}^{2}\right)\right].$$

The computation of importance values requires an additional pass over the training data D and the estimation of the log probabilities log*p*_**Θ**_ represented by the network outputs. Following Schwarz et al. (2018), we keep a single importance matrix for all steps, by summing the importance on the current step with the previous values. In order to prevent the unbounded growth of importance values we normalize between 0 and 1 when computing importance on the current step.

#### 2.1.2. Learning Without Forgetting

Learning without Forgetting (LwF) (Li and Hoiem, 2016) is a regularization technique which preserves the knowledge of previous steps by fostering stability at the activation level through knowledge distillation (Hinton et al., 2015). The method adds a regularization term R to the loss during step *n* as follows:


(3) $$ℛ(\Theta_{n},\Theta_{n-1};x,y)=\alpha \text{ KL}[p_{\Theta_{n}}(y|x)\;||\;p_{\Theta_{n-1}}(y|x)],$$

where α controls the regularization strength. The KL-divergence term prevents current activations to diverge too much from the ones of the model at previous step.

#### 2.1.3. Replay

Replay of previous patterns during training is a very effective technique against forgetting of existing knowledge (Hayes et al., 2018; Aljundi et al., 2019; Chaudhry et al., 2019b; Rolnick et al., 2019). We leveraged a replay memory which stores a fixed number of patterns for each class. During training on each step, the replay memory is concatenated with the training set. The resulting dataset is shuffled and used for training the model. Therefore, replay patterns are spread uniformly over the training set.

#### 2.1.4. Naïve

The Naïve strategy trains the model continuously without applying any CL technique. This strategy is heavily subjected to catastrophic forgetting. Therefore, it can be used as a baseline to compare the performance of more effective CL strategies, which should perform significantly better in terms of forgetting.

### 2.2. Deep Graph Networks Models

We define a graph as a tuple $g=\left(V_{g},E_{g},X_{g},A_{g}\right)$ where $V_{g}$ is the set of *nodes*, $E_{g}$ is the set of oriented *edges* connecting ordered pairs of nodes, whereas $X_{g}$ (respectively $A_{g}$) denotes node (edge) features. The neighborhood $N_{v}$ of a node *v* is the set of all nodes *u* for which an edge (*u, v*) directed toward *v* exists.

#### 2.2.1. Structure-Agnostic Baseline

To assess whether continual learning strategies have an impact when working with graphs, we must first devise a baseline that ignores the structural information and relies only on node features. The most common baseline we find in the literature (Dwivedi et al., 2020; Errica et al., 2020) is a multi-layer perceptron (MLP) that is invariant to the ordering of the nodes. Formally, the baseline compute a node representation **h**_*v*_ as follows


(4) $$h_{v}=\psi (x_{v}),\;x_{v}\in X_{g},$$

(5) $$\psi (x_{v})=W_{L}^{T}(\sigma (\ldots (\sigma (W_{1}^{T}x_{v}+b_{1})\ldots )+b_{L}),$$

where ψ(·) is an MLP of *L* layers, the symbol **W** denotes a weight matrix and **b** is the bias. As the tasks under consideration in this paper deal with graph classification, an additional *readout* phase is necessary, in which we aggregate all node representations into a single graph representation **h**_*g*_:


(6) $$h_{g}=\Psi_{g}(\{h_{v}|v\in V_{g}\}),$$

where Ψ_*g*_ is a permutation invariant function; in this work we will use the *mean* function as the baseline's readout.

#### 2.2.2. Deep Graph Networks

While DGNs usually adopt the same readout scheme as the one of Equation 6, the fundamental difference lies in its graph convolutional layer. If we assume a deep network of *L* layers, the node representation at layer ℓ < *L*, that is, ${\text{h}}_{v}^{\ell }$ is obtained by aggregating the neighboring information of all nodes using another permutation invariant function Ψ_*n*_:


(7) $$h_{v}^{\ell +1}=\phi^{\ell +1}(h_{v}^{\ell },\;\Psi_{n}(\{\psi^{\ell +1}(h_{u}^{\ell })|u\in N_{v}\})),$$

where ϕ and ψ are usually implemented as linear layers or MLPs.

In our experiments, we define Ψ_*n*_ as the *mean* operator for digit classification tasks and sum for the chemical ones.

#### 2.2.3. Structure-Preserving Regularization Loss

We believe it is worth investigating whether a structure-preserving regularization loss such as the one of Kipf and Welling (2017) affects catastrophic forgetting when used alongside the various CL strategies. The catch is that regularization will help preserve the output of previously seen classes when similar structural patterns appear in the new training samples. In general, the interplay between GRL and CL regularization strategies opens appealing research directions for the future. In case the chosen regularization does not help, this may indicate that the distribution of neighbor states of patterns belonging to a new class is radically different from those seen before.

## 3. Results

This section provides a thorough description of the experimental details necessary to reproduce our experiments and of the results we obtained. The code is made publicly available to reproduce the results and carry out novel robust evaluations of different continual learning strategies^2^.

In all our experiments, we performed model selection on the validation set using a grid-search strategy for all the implemented models. Regardless of the dataset or continual learning technique used, we selected the number of layers in {2, 4} for the DGN and 4 for the baseline. In both cases, the dimension of the hidden layer was chosen in {64, 128}. The number of epochs was set to 200 (patience = 20) for the Baseline and to 1,000 for DGN and DGN+Reg (patience = 50). The learning rate was set to 0.001, and the optimizer chosen was Adam. We used the “sum” version of the EWC combined with normalized importance scores. Being LWF very sensible to the hyper-parameters, we chose α∈{0.5, 1.0, 2.0} and the temperature in {0.5, 1.0, 2.0}.

### 3.1. Datasets

The evaluation is carried out on three different large graph classification datasets. The former two, namely MNIST and CIFAR10, are the standard digit classification benchmarks used in the CL literature. However, here the digits are represented as graphs of varying dimension and shape (Dwivedi et al., 2020). The nodes are “superpixels” obtained through a specific coarsening process, and the adjacency information is constructed using the *k*-nearest neighbor algorithm. We defer the specifics of this process to the original paper. The third dataset is OGBG-PPA (Hu et al., 2020), a dataset of undirected protein association neighborhoods taken from protein-protein interaction graphs. Here, the task is to classify each input as one of 37 different taxonomy groups. Here, node features are missing but edges contain information. As such, we treat edges as nodes in the structure-agnostic baseline. We use the same data splits as those provided in the original papers, thus performing standard hold-out model selection and assessment. We also use the readily available version of all datasets provided by the Pytorch Geometric library (Fey and Lenssen, 2019). Table 1 summarizes some useful dataset statistics.

**Table 1:** Summary of the datasets statistics.

	**MNIST**	**CIFAR10**	**OGBG-PPA**
Size	70,000	60,000	158,100
Node attrs.	3	5	0
Edge attrs.	0	0	7
Classes	10	10	37
Avg $\left|V_{g}\right|$	70.57	117.63	243.4
Avg $\left|E_{g}\right|$	564.63	941.07	2266.1
Data split	55K/5K/15K	45K/5K/15K	49%/29%/22%
Class split	2+2+2+2+2	2+2+2+2+2	17+5+5+5+5

“*Class split” refers to how we group classes in the Split CL experiment*.

### 3.2. Continual Learning Evaluation Protocol

We evaluated each model in the class-incremental scenario, a popular continual learning setting where new classes arrive over time (van de Ven and Tolias, 2018). When a new step arrives, the model is trained on the new data without using data from the previous steps (except for the replay buffer, when used). We use single-head models, where the entire output layer is used at each step. This is one of the most challenging scenarios for the mitigation of catastrophic forgetting in CL. Table 1 shows the class splits for each dataset, highlighting how many new classes are present in each step. We monitor the metric $\text{ACC}=\frac{1}{T}\overset{T}{\underset{t=1}{\sum }}R_{T,t}$, introduced in Lopez-Paz and Ranzato (2017), where *R*_*T, t*_ is the accuracy on step *t* after training on step *T*.

We reported the average ACC and its standard deviation computed over 5 runs. Larger final accuracy corresponds to a smaller degree of forgetting, sometimes also referred to as Negative Backward Transfer in the continual learning literature (Lopez-Paz and Ranzato, 2017). We evaluated the performance by computing the mean accuracy over all the steps after training on all steps.

### 3.3. Catastrophic Forgetting Analysis

**Figure 1:**
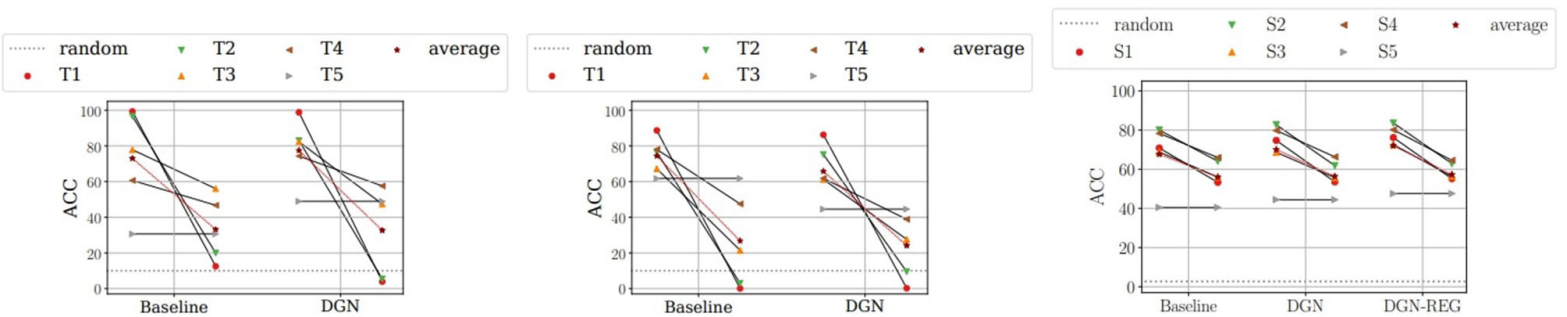
Paired plots showing the ACC on each step for different models for LWF and MNIST **(left)**, LWF and CIFAR10 **(middle)**, Replay and OGBG-PPA **(right)**. Complete plots in the Supplementary Material. Each column refers to a model and it is composed by pairs of connected points. Each pair refers to a specific step. The leftmost point in the pair represents ACC after training on that specific step. The rightmost point represents ACC after training on all steps. The more vertical the line connecting the points, the larger the forgetting effect. The dashed horizontal line indicates the performance of a random classifier. The red star represents the average performance over all steps.

The empirical results suggest that Deep Graph Networks trained continuously are subjected to catastrophic forgetting of previous knowledge. Table 2 reports the average ACC across all steps (see also Figure 1 for an intuitive visualization of results). We also extend the results presented in Lesort et al. (2020) to Deep Graph Networks: importance-based regularization strategies are not able to prevent forgetting in class-incremental scenarios. In fact, in our experiments EWC always performs comparably to the *Naïve* strategy.

**Table 2:** Mean accuracy and mean standard deviation among all steps.

	**Model**	**Strategy**			
		**Naïve**	**EWC**	**Replay**	**LWF**
MNIST	Baseline	19.56 ± 0.1	19.39 ± 0.1	86.13 ± 4.5	33.16 ± 13.1
	DGN	19.19 ± 0.1	18.95 ± 0.3	79.52 ± 1.9	32.64 ± 5.0
	DGN+reg	19.31 ± 0.1	—	81.42 ± 2.4	—
CIFAR10	Baseline	17.49 ± 0.1	17.49 ± 0.1	42.87 ± 3.7	26.77 ± 5.1
	DGN	17.11 ± 0.2	17.10 ± 0.2	39.55 ± 2.3	24.13 ± 4.1
	DGN+reg	17.13 ± 0.1	—	46.61 ± 3.5	—
OGBG-PPA	Baseline	14.53 ± 0.5	13.90 ± 0.8	55.96 ± 3.0	20.83 ± 6.1
	DGN	14.47 ± 0.3	14.15 ± 0.5	56.34 ± 2.5	18.46 ± 5.4
	DGN+reg	15.18 ± 0.8	—	57.27 ± 3.2	—

*Smaller accuracy results in larger forgetting of previous knowledge. Replay results are related to memory size of 1, 000. Results are averaged over 5 final runs. We treat the regularization loss as a separate strategy*.

Interestingly, Deep graph networks do not provide significant performance improvements with respect to a structure-agnostic baseline. This is a surprising result, which might have two complementary explanations. The first is that the neighboring states' distribution of different classes varies, thus making the previously trained graph convolutions inadequate for subsequent tasks. The second, instead, relates to the nature of the class-incremental scenario. Since the model sees few classes at a time, each training task becomes so simple that the model ends up relying on node features only to discern between the two classes. This is confirmed by the fact that, when encouraged to retain structural information *via* the regularization term, DGN shows a slight increase in performance with the replay strategy. We believe that addressing both points in more detail could constitute interesting future work at the intersection of the two research fields.

#### 3.3.1. Sensitivity of LwF to Hyperparameters

**Figure 2:**
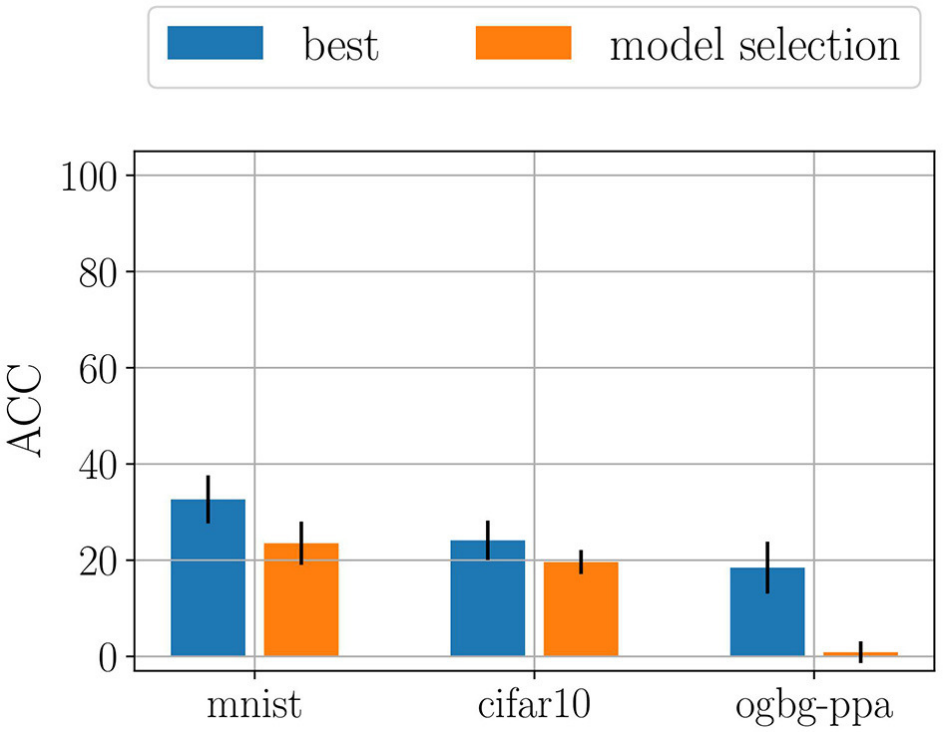
Comparison of performances between model selection (averaged across all configurations) and model assessment (averaged across 5 final training runs). The difference highlights the sensitivity of LwF to the choice of hyperparameters. Most of the configurations cause larger forgetting effects with respect to the best configuration found by model selection.

Not all regularization strategies are, however, subjected to forgetting. In fact, we show that LwF is able to recover part of the original knowledge, outperforming both Naïve and EWC. We also found LwF to be very sensitive to the choice of the hyperparameters. In particular, the softmax temperature and the hyperparameter α, which controls the amount of knowledge distillation heavily influence the final performance. In order to best show the sensitivity of LwF to the choice of hyperparameters, we computed the mean ACC and its standard deviation across all runs of model selection (Figure 2). Then, we compared the results with the best performance we found during model assessment. The difference highlights the high sensitivity of Lwf which could partially limit its applicability in real world applications, where it may be impossible to perform appropriate model selection in continual learning scenarios (Chaudhry et al., 2019a).

#### 3.3.2. Effectiveness of Replay

Replay strategy is considered among the strongest CL strategies available. In our experiments, replay consistently outperforms all the other strategies. Supplementary Material shows ACC values for increasing replay memory sizes. Deep graph networks and baseline models require a comparable amount of replay to obtain the same level of performance. Therefore, replay seems to behave as a good model-agnostic strategy even in the domain of graphs.

## 4. Discussion

Learning from a data stream in a continual fashion is a key property of many biological systems, including the human brain. In fact, continual learning is often considered as a necessary condition for the development of artificial intelligent agents operating in the real world. Even though there exist continual learning strategies loosely inspired by neuroscience, most of them do not take into consideration that we, as humans, act in a world filled with structured and complex interactions between different objects. On one side, applying graph representation learning techniques to continual learning may lead to the acquisition of durable knowledge, since each piece of information is related to many others and it may be therefore more difficult to forget it. On the other side, our results highlighted that before being able to achieve this objective, it will be necessary to design *ad-hoc* continual learning strategies which explicitly take into consideration the structure and relations present in the data.

Our empirical evaluation of continual learning strategies with graph-structured data focused on the catastrophic forgetting phenomenon which affects deep graph networks in class-incremental scenarios. We evaluated a number of existing CL approaches and we discussed whether they are able to retain previous knowledge when applied to deep graph networks.

Interestingly, while graph networks outperform feedforward baselines during offline training, our results show that this advantage disappears in continual learning scenarios. This suggests that structure-preserving regularization techniques may help DGNs to mitigate catastrophic forgetting. Nonetheless, the results are still far from the performance achieved in the offline setting, where all data is available at the beginning of training. This can be easily seen by looking at the performance of the replay strategy, which largely improves over all the other strategies. Since replay approximates the offline training regime for large replay memory sizes, its performance can be considered as an upper-bound for the other continual learning strategies. Unfortunately, storing previous patterns is not always possible in real-world environments (e.g., due to privacy reasons or memory constraints). The design of *ad-hoc* DGNs and regularization techniques constitutes a valid, replay-free alternative. However, it would be interesting to limit the disadvantages of replay without sacrificing its performance. For example, latent replay approaches do not store raw input patterns to rehearse previous knowledge, but only latent and hidden activations of the model. In the presence of graph-structured data, storing few activations may be enough to reconstruct significant portions of the others. In the future, as supported by Hayes et al. (2021), latent replay on structured data can be empowered by better understanding the role played by replay in biological systems and in the human brain, where partial stimuli are often sufficient to reconstruct previous experiences in detail.

By releasing the code of our experiments, and by providing a robust evaluation protocol for continual learning on some graph classification tasks, we hope to contribute to further progresses in the understanding of how novel continual learning strategies can be applied to the domain of graphs.

## Data Availability Statement

The original contributions presented in the study are included in the article/Supplementary Material, further inquiries can be directed to the corresponding author/s.

## Author Contributions

ACa, ACo, and FE contributed to the definition of the benchmark, the experimental setup, and the evaluation of the experiments. DB contributed to writing the paper and to provide feedbacks on the future impacts of the work. All authors contributed to the article and approved the submitted version.

## Funding

This work has been partially supported by the European Community H2020 programme under project TEACHING (Grant No. 871385).

## Conflict of Interest

The authors declare that the research was conducted in the absence of any commercial or financial relationships that could be construed as a potential conflict of interest.

## Publisher's Note

All claims expressed in this article are solely those of the authors and do not necessarily represent those of their affiliated organizations, or those of the publisher, the editors and the reviewers. Any product that may be evaluated in this article, or claim that may be made by its manufacturer, is not guaranteed or endorsed by the publisher.

## Footnotes

^1^This term disambiguates the more common “Graph Neural Networks” (GNN), which refers to the work of Scarselli et al. (2009).

^2^ https://github.com/diningphil/continual_learning_for_graphs
