# Supplementary material for: Catastrophic Forgetting in Deep Graph Networks: A Graph Classification Benchmark
Source: Front Artif Intell. 2022 Feb 4;5:824655. doi: 10.3389/frai.2022.824655 (PMC8855050; doi:10.3389/frai.2022.824655)
Supplement: Supplementary file 1 [file Presentation_1.pdf]

Supplementary Material

1 REPLAY PLOTS

Figure S1 shows the performance of replay strategies for increasing replay memory sizes.

2 VISUALIZATION OF RESULTS WITH PAIRED PLOTS

We report the complete set of paired plots produced during our experiments (Figure S2 to Figure S13). As described in the main text, each column in the plot refers to a model and it is composed by pairs of connected points. Each pair refers to a specific step. The leftmost point in the pair represents ACC after training on that specific step. The rightmost point represents ACC after training on all steps. The more vertical the line connecting the points, the larger the forgetting effect. The dashed horizontal line indicates the performance of a random classifier. The red star represents the average performance over all steps.

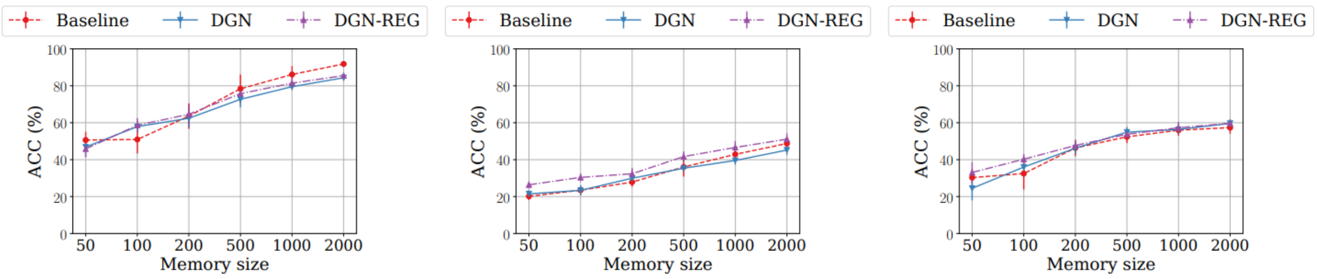

Figure S1. ACC for increasing replay memory size for MNIST (left), CIFAR-10 (middle) and OGBG-PPA (right).

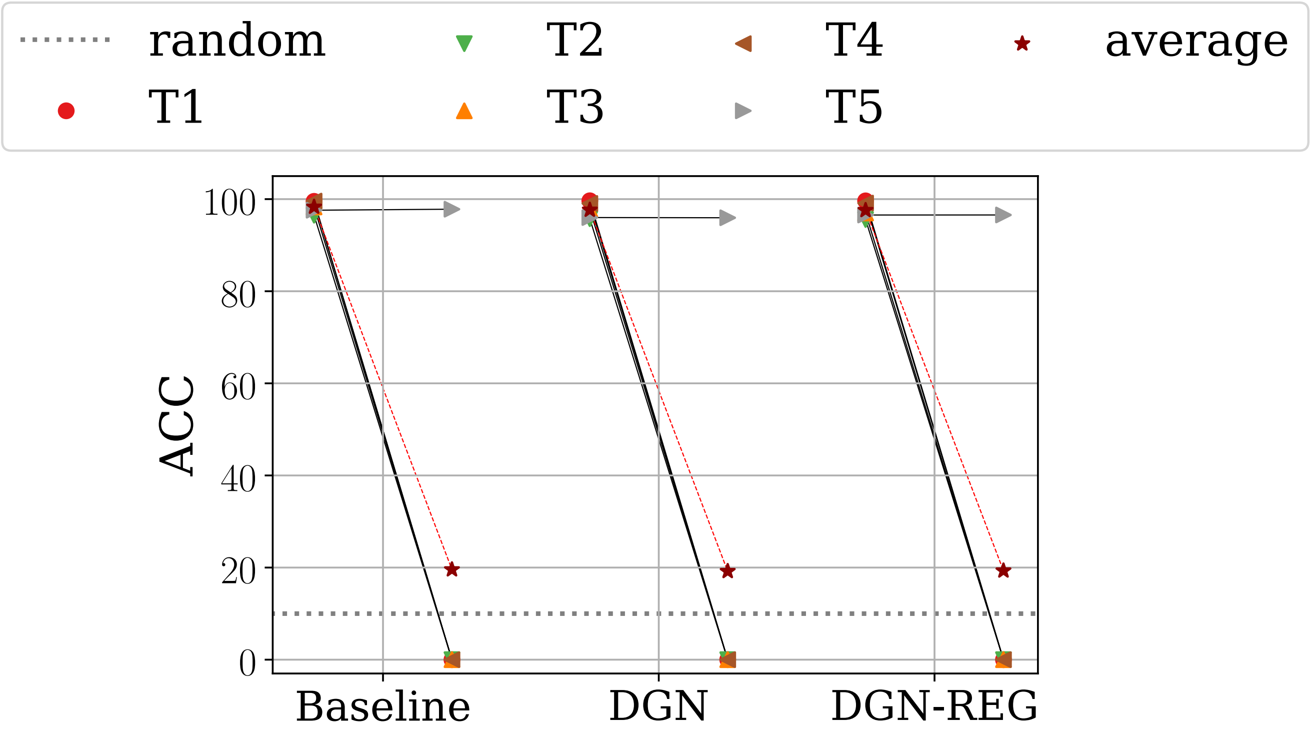

Figure S2. MNIST + Naïve

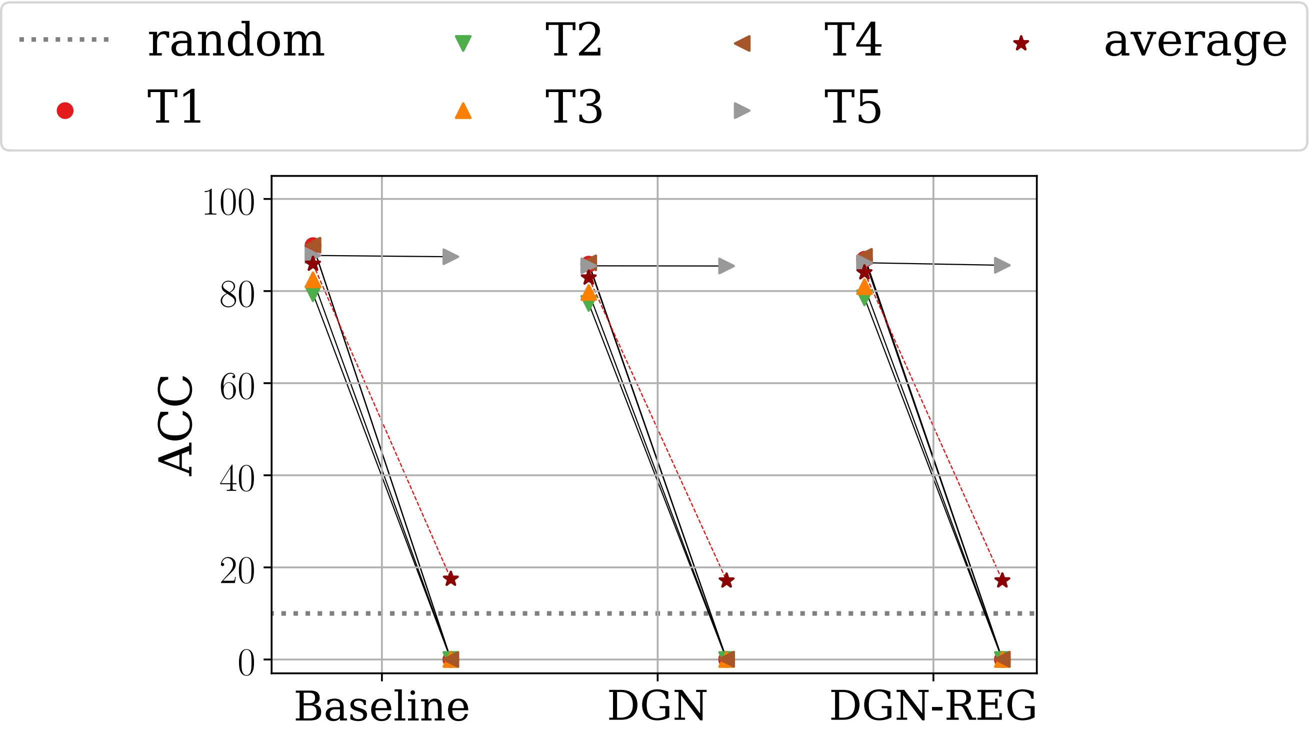

Figure S3. CIFAR10 + Naïve

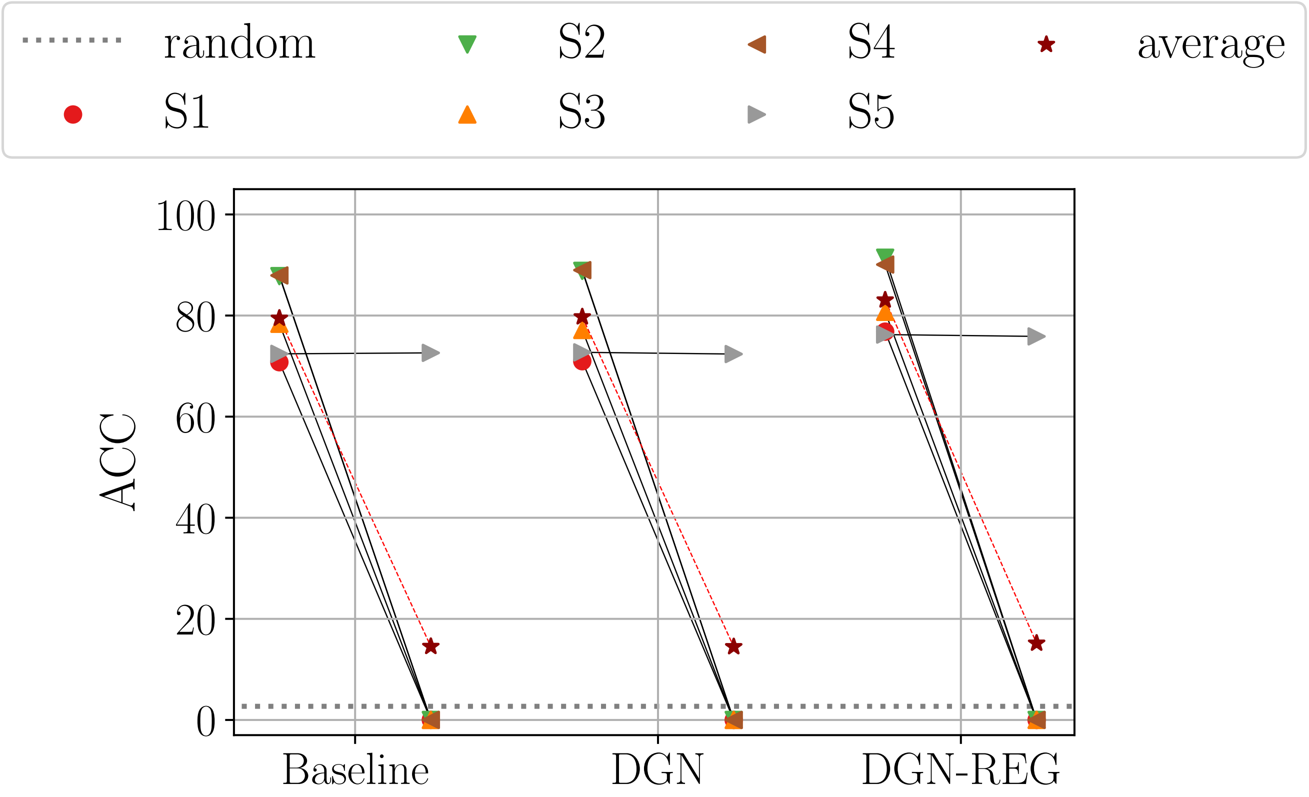

Figure S4. OGBG-PPA + Naïve

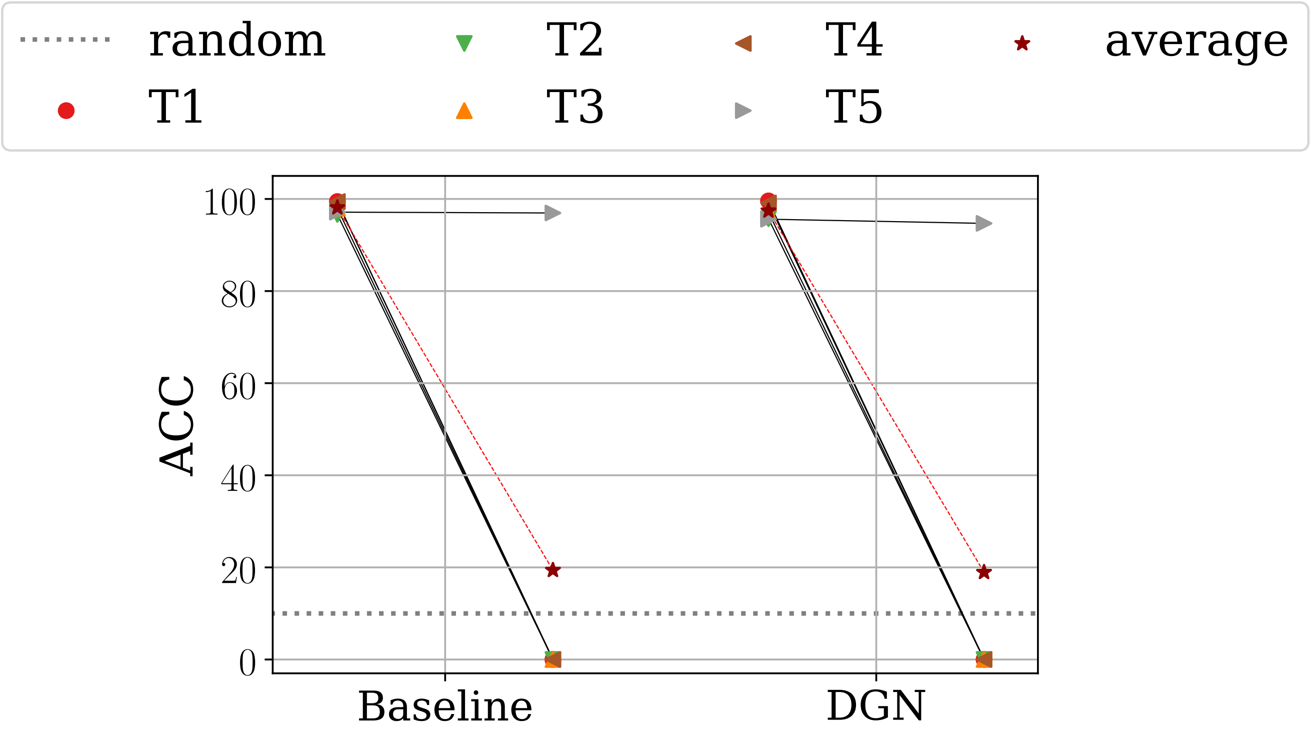

Figure S5. MNIST + EWC

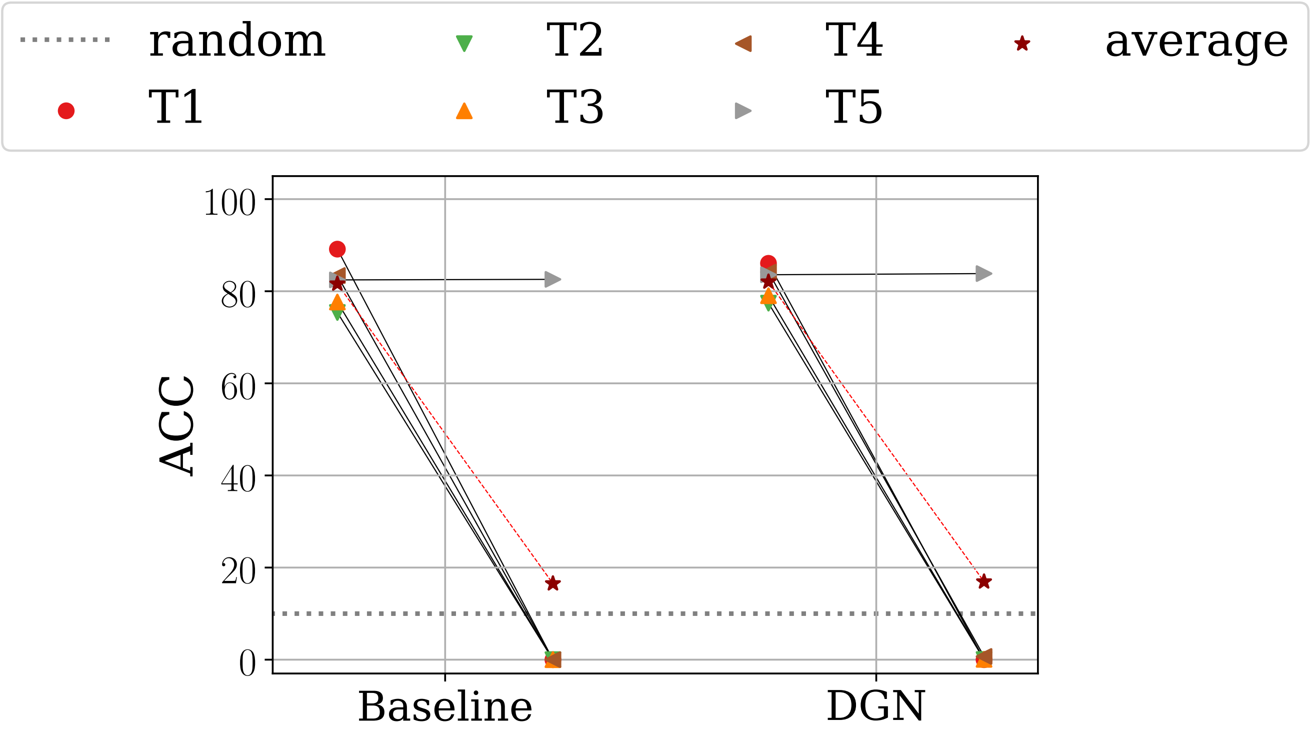

Figure S6. CIFAR10 + EWC

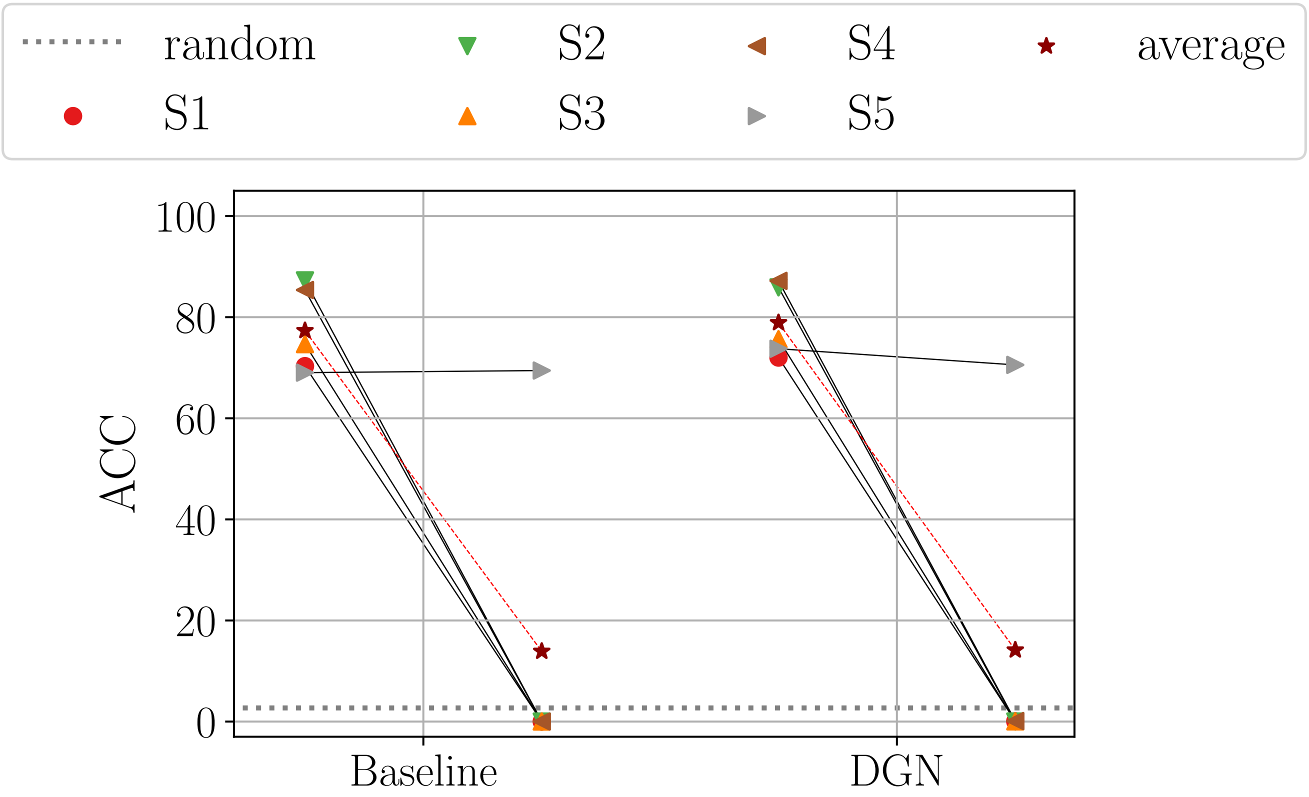

Figure S7. OGBG-PPA + EWC

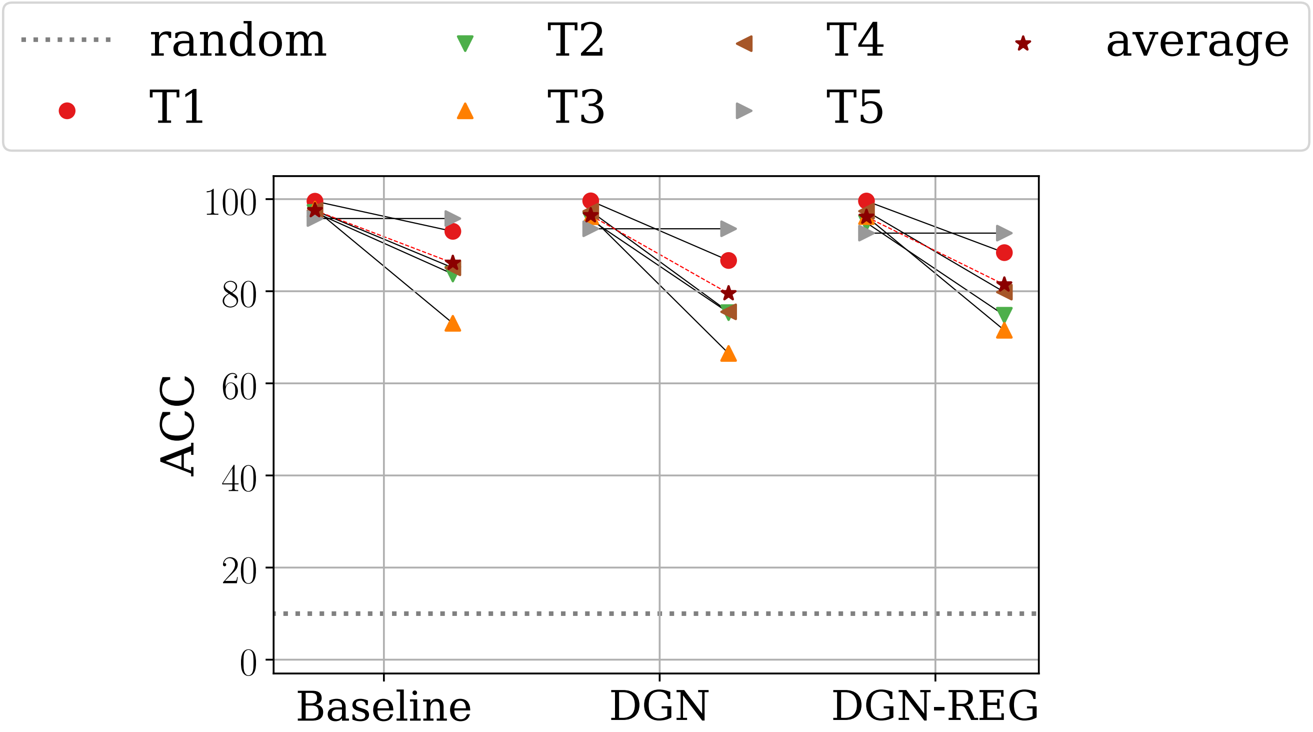

Figure S8. MNIST + REPLAY

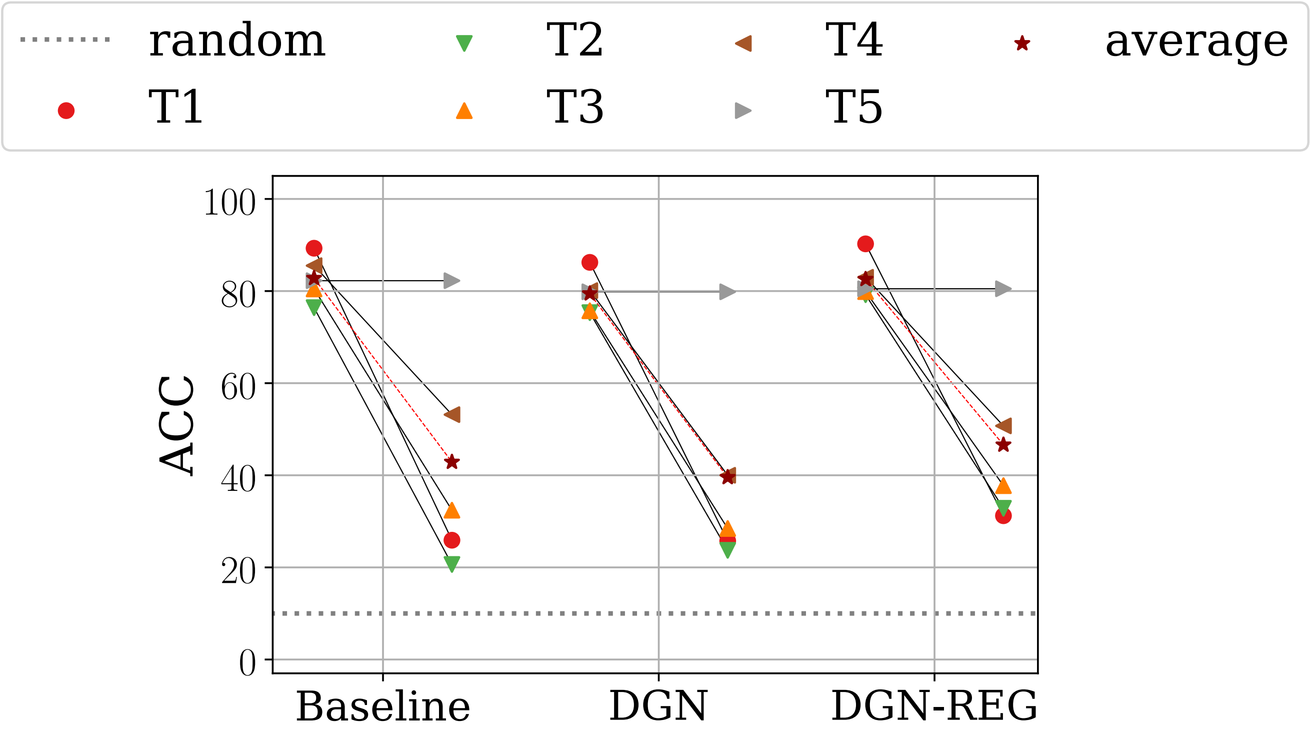

Figure S9. CIFAR10 + REPLAY

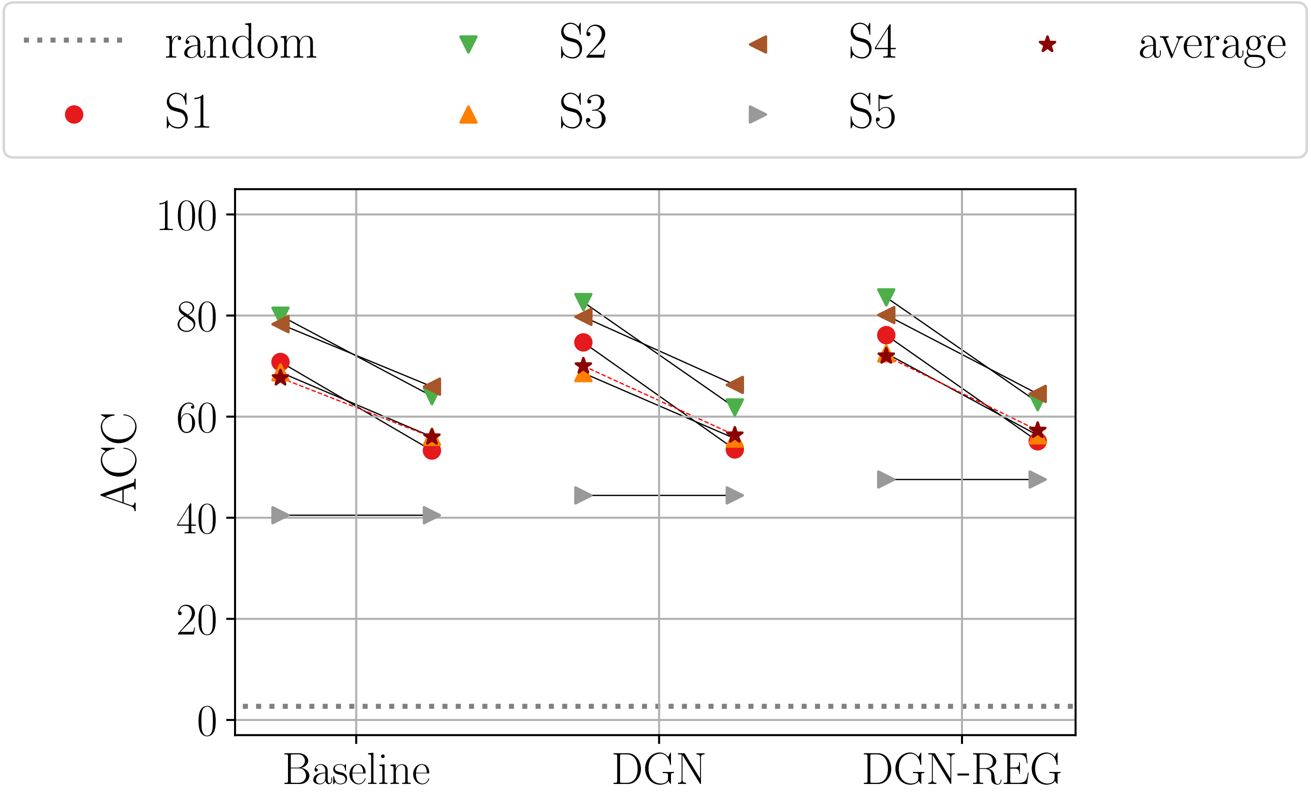

Figure S10. OGBG-PPA + REPLAY

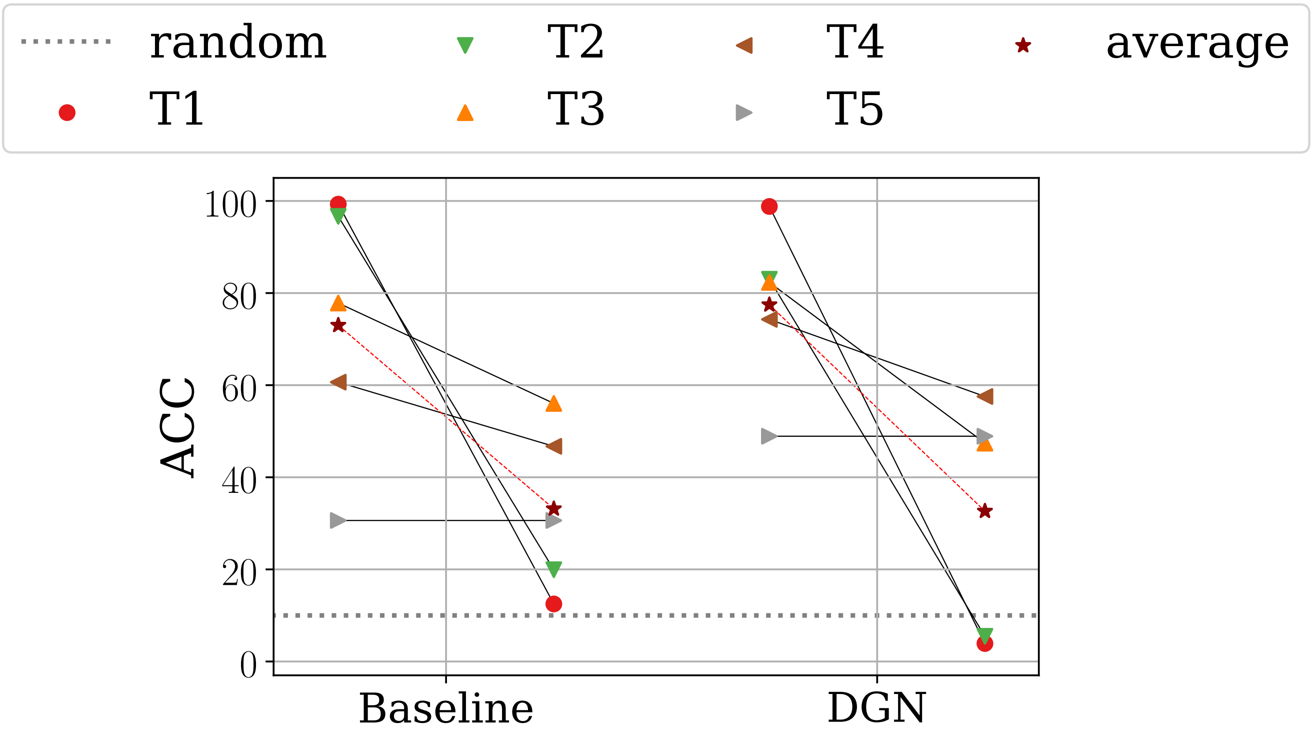

Figure S11. MNIST + LwF

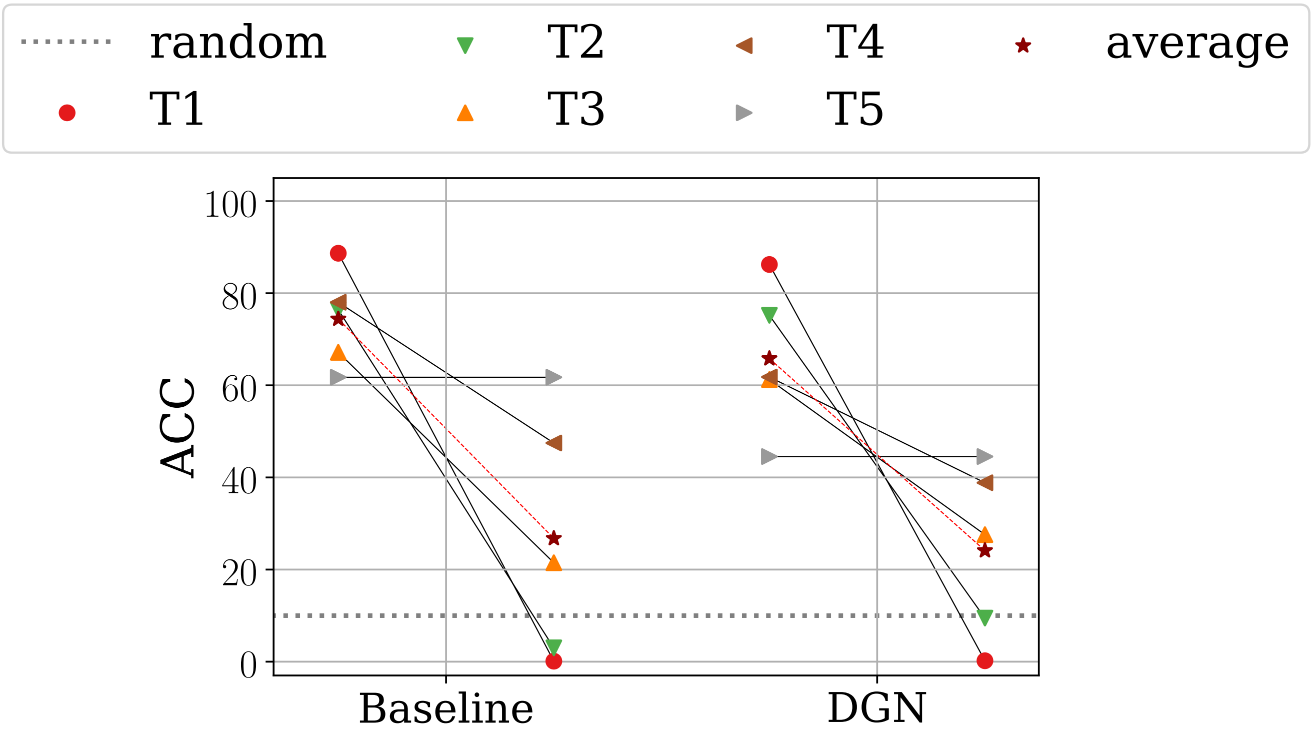

Figure S12. CIFAR10 + LwF

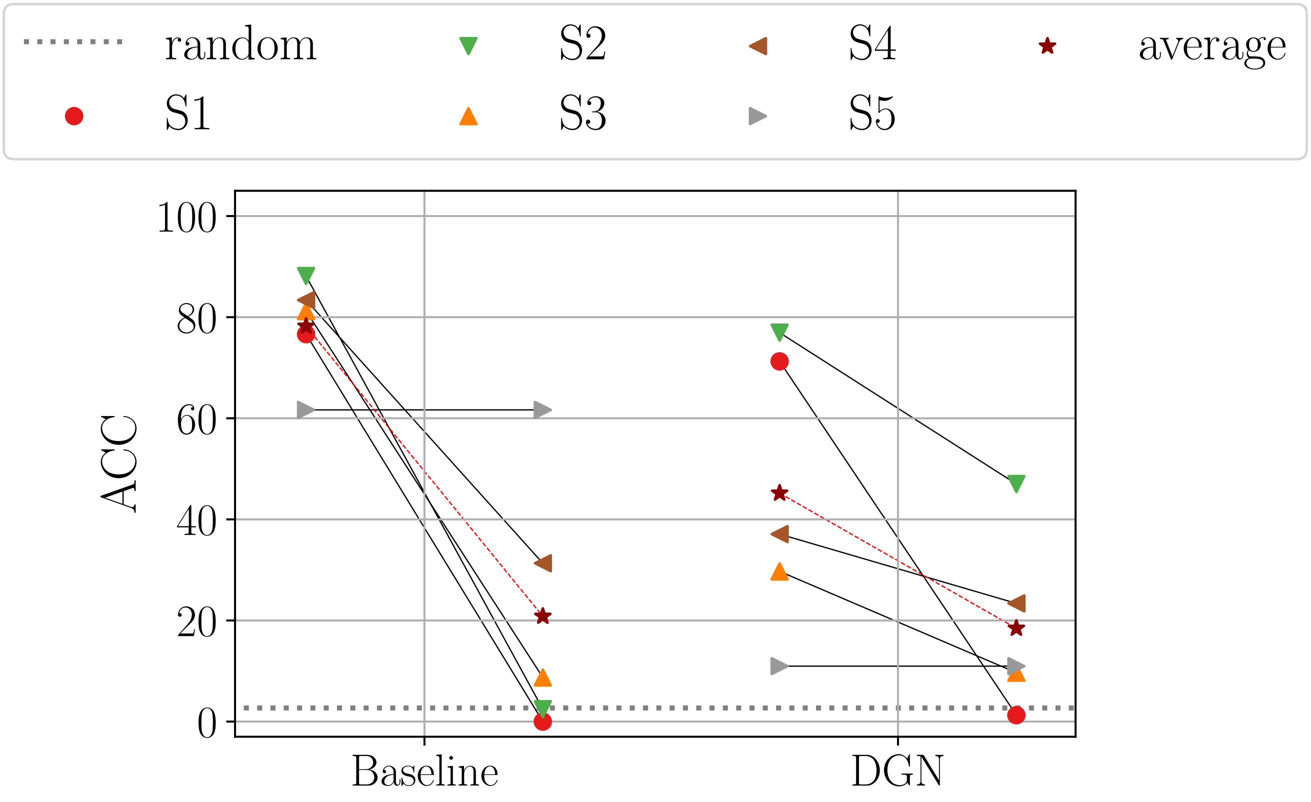

Figure S13. OGBG-PPA + LwF
